# Functional overexpression of genes involved in erythritol synthesis in the yeast *Yarrowia lipolytica*

**DOI:** 10.1186/s13068-017-0772-6

**Published:** 2017-03-24

**Authors:** Aleksandra M. Mirończuk, Anna Biegalska, Adam Dobrowolski

**Affiliations:** Department of Biotechnology and Food Microbiology, Wroclaw University of Environmental and Life Sciences, Chełmońskiego 37, 51-630 Wrocław, Poland

**Keywords:** *Yarrowia lipolytica*, Erythritol, Glycerol, Transketolase, Pentose phosphate pathway

## Abstract

**Background:**

Erythritol, a four-carbon polyol synthesized by microorganisms as an osmoprotectant, is a natural sweetener produced on an industrial scale for decades. Despite the fact that the yeast *Yarrowia lipolytica* has been reported since the 1970s as an erythritol producer, the metabolic pathway of this polyol has never been characterized. It was shown that erythritol synthesis in yeast occurs via the pentose phosphate pathway (PPP). The oleaginous yeast *Y. lipolytica* is a good host for converting inexpensive glycerol into a value-added product such as erythritol. Glycerol is a renewable feedstock which is produced on a large scale as a waste product by many branches of industry.

**Results:**

In this study, we functionally overexpressed four genes involved in the pentose phosphate pathway (PPP): gene *YALI0E06479*g encoding transketolase (*TKL1*), gene *YALI0F15587*g encoding transaldolase (*TAL1*), gene *YALI0E22649*g encoding glucose-6-phosphate dehydrogenase (*ZWF1*), and gene *YALI0B15598*g encoding 6-phosphogluconate dehydrogenase (*GND1*). Here, we show that the crucial gene for erythritol synthesis in *Y. lipolytica* is transketolase. Overexpression of this gene results in a twofold improvement in erythritol synthesis during a shake-flask experiment (58 g/L). Moreover, overexpression of *TKL1* allows for efficient production of erythritol independently from the supplied dissolved oxygen. Fermentation conducted in a 5-L bioreactor at low agitation results in almost 70% higher titer of erythritol over the control strain.

**Conclusion:**

This work presents the importance of the PPP in erythritol synthesis and the feasibility for economic production of erythritol from glycerol by the yeast *Y. lipolytica*.

**Electronic supplementary material:**

The online version of this article (doi:10.1186/s13068-017-0772-6) contains supplementary material, which is available to authorized users.

## Background

Erythritol is a well-known natural sweetener, commonly occurring in fruits, wine, and honey [1]. This four-carbon compound belongs to the group of polyols, the so-called sweet alcohols. Erythritol does not change the insulin level in blood; thus, it can be used by diabetics. Due to its impressive properties such as low energy value (~0.2 kcal/g), natural sweet taste (70% of sucrose sweetness), and the highest no-effect dose for causing diarrhea among the polyols [2], erythritol has been produced for decades. On the industrial scale, erythritol is produced in aerobic fermentation processes using *Torula* sp. [3] and *Moniliella* sp. [4]. Moreover, other microorganisms such as the yeast *Yarrowia lipolytica* are also able to produce erythritol [5].

Erythritol is produced as an osmoprotectant by osmophilic yeast and some bacteria as a cell response to high osmotic pressure of the environment. This feature is used in industry, where highly osmotic media (up to 40% of glucose) are applied to stimulate erythritol synthesis.

Many studies have been performed to obtain high-production organisms and to improve fermentation conditions for high productivity [6–9]. However, the most important issue in polyol synthesis is the high-production costs (caused by expensive media), oxygen supply and a high content of byproducts such as glycerol, ribitol, mannitol, and organic acids. For economic reasons, an alternative, low-value carbon source for erythritol biosynthesis is sought. Good substitutes of glucose for industrial applications are agricultural wastes, biodiesel byproducts, or other renewable feedstocks such as glycerol. Every year, crude glycerol is produced in huge quantities by many industrial branches, e.g., in biodiesel production, fat saponification, stearin production, and alcoholic beverage production units [10]. Despite the fact that crude glycerol is contaminated by many undesired compounds such as methanol, salts, and matter organic non-glycerol (MONG), some microorganisms are capable of metabolizing it; one of them is *Y. lipolytica* [11]. The unconventional yeast *Y. lipolytica* is a well-studied oleaginous model organism for investigating lipid production and accumulation in eukaryotic cells. However, it also possesses capability for production of polyols and organic acid [12–18]. Despite many studies on erythritol synthesis using *Y. lipolytica*, the metabolic pathway of this phenomenon has never been described. It was shown that in fungus and yeast, erythritol synthesis occurs via the pentose phosphate pathway (PPP) [19, 20] and in the final step, erythrose reductase (ER) reduces erythrose to erythritol with concomitant NAD(P)H oxidation [1].

The aim of this study was to identify the key genes involved in erythritol synthesis. In this study, we overexpressed genes involved in PPP, transketolase (*TKL1*, *YALI0E06479*g), transaldolase (*TAL1*, *YALI0F15587*g), and two dehydrogenases: *ZWF1* (*YALI0E22649*g) and *GND1* (*YALI0B15598*g), responsible for production of reducing agents in the cell, to verify their roles in erythritol biosynthesis. Moreover, we found that the engineered strains are able to produce erythritol at low aeration, this being beneficial for downstream processing. The gene which has the highest impact on erythritol synthesis is transketolase.

## Methods

### Microorganisms, media, and culture conditions

The *Y. lipolytica* strains used in this study were derived from the strain *Y. lipolytica* MK1 [21]. All the strains used in this study are listed in Table 1. In Table 2, the gene name, gene symbol, and abbreviation are summarized.

**Table 1:** Strains and plasmids used in this study

Strain	Genotype or plasmid	Source
*E. coli*		
DH5α	F^−^ endA1 glnV44 thi-1 recA1 relA1 gyrA96 deoR nupG Φ80dlacZΔM15 Δ(lacZYA-argF)U169, hsdR17(rK-mK+), *λ*−	[33]
DH5α	pAD, UAS1_B16_TEF promoter	This study
DH5α	pAD-TKL1, *YALI0E06479*g	This study
DH5α	pAD-TAL1, *YALI0F15587g*	This study
DH5α	pAD-GDN1, *YALI0B15598*g	This study
DH5α	pAD-ZWF1, *YALI0E22649*g	This study
Dh5 α	phrGFP II-C	Agilent
Dh5 α	pAD-hrGFP	This study
Dh5 α	P_TKL1_-gfp	This study
Dh5 α	P_TAL1_-gfp	This study
Dh5 α	P_GDN1_-gfp	This study
Dh5 α	P_ZWF1_-gfp	This study
*Y. lipolytica*		
AMM	*MATA*, MK1: ura3-302	[28]
MK1	*MATA, UV-mutant*	[21]
AMM pAD-TKL1	*MATA*, MK1: ura3-302, overexpression *YALI0E06479*g	This study
AMM pAD-TAL1	*MATA*, MK1: ura3-302, overexpression *YALI0F15587g*	This study
AMM pAD-GDN1	*MATA*, MK1: ura3-302, overexpression *YALI0B15598*g	This study
AMM pAD-ZWF1	*MATA*, MK1: ura3-302, overexpression *YALI0E22649*g	This study
AMM P_TKL1_-gfp	*MATA*, MK1: ura3-302, promoter TKL1 fused to hr*gfp*	This study
AMM P_TAL1_-gfp	*MATA*, MK1: ura3-302, promoter TAL1 fused to hr*gfp*	This study
AMM P_GDN1_-gfp	*MATA*, MK1: ura3-302, promoter GDN1 fused to hr*gfp*	This study
AMM P_ZWF1_-gfp	*MATA*, MK1: ura3-302, promoter ZWF1 fused to hr*gfp*	This study

**Table 2:** Description of genes overexpressed in *Y. lipolytica*

Gene	Number	Function
*TKL1*	YALI0E06479g	Transketolase
*TAL1*	*YALI0F15587*g	Transaldolase
*ZWF1*	*YALI0E22649*g	Glucose-6-phosphate dehydrogenase
*GND1*	*YALI0B15598*g	6-Phosphogluconate dehydrogenase

*Escherichia coli* strains were cultivated in LB medium according to standard protocols. Rich yeast extract peptone glucose (YPD) medium was used for the yeast inoculum preparation and contained 1% (w/v) yeast extract, 1% (w/v) peptone, and 2% (w/v) glucose.

During shake-flask experiments, the cultures were grown in 0.3-L Erlenmeyer flasks or 0.25-L baffled flasks containing 0.03 L medium on a rotary shaker (CERTOMAT IS, Sartorius Stedim Biotech) at 28 °C at 280 rpm. Erythritol synthesis was conducted in Erythritol Synthesis Medium (g/L): 100 glycerol (Chempur, Poland), 2.3 (NH_4_)_2_SO_4_ (Chempur), 1 MgSO_4_ × 7H_2_O (Chempur), 0.23 KH_2_PO_4_ (Chempur), NaCl 26.4 (Chempur), 1 yeast extract (Merck, Germany), and 3 CaCO_3_, pH 3.0.

The Control Medium was as follows: (g/L): 100 glucose (Merck, Germany), 2.3 (NH_4_)_2_SO_4_ (Chempur), 1 MgSO_4_ × 7H_2_O (Chempur), 0.23 KH_2_PO_4_ (Chempur), NaCl 26.4 (Chempur), 1 yeast extract (Merck), and 3 CaCO_3_, pH 3.0.

### Bioscreen C

The yeast strain was grown in 100-well plates in 200 μL of YNB supplemented with glycerol 2% (w/v) or glucose 2% (w/v). First, the strains were grown for 24 h in YPD medium containing 1% (w/v) yeast extract, 1% (w/v) peptone, and 2% (w/v) glucose. The cells were washed and inoculated to an OD_600_ of 0.15 in each well. Quintuple experiments were performed at 28 °C under constant agitation with a Bioscreen C (Oy Growth Curves Ab Ltd., Finland). Growth was monitored by measuring the optical density at 420–560 nm every 30 min for 72 h.

### Bioreactor studies

To prepare an inoculation culture for fermentation in a bioreactor, the cultures were grown in 0.3-L Erlenmeyer flasks (containing 0.1 L of YPD medium) on a shaker at 28 °C for 72 h at 140 rpm. Erythritol production was conducted in a medium consisting of (g/L): 150 glycerol, 2.3 (NH_4_)_2_SO_4_, 1 MgSO_4_ × 7H_2_O, 0.23 KH_2_PO_4_, NaCl 26.4, and 1 yeast extract, pH 3.0. An inoculum of 0.2 L was introduced into the bioreactor containing the production medium. The cultivations were performed in a 5-L jar bioreactor (Biostat B Plus, Sartorius, Germany) with a working volume of 2 L at 28 °C. The aeration was fixed at 0.8 L/min. The stirrer speed was adjusted to 500 rpm. The pH was maintained automatically at 3.0 via the addition of NaOH (40% w/v). The amount of the supplied NaOH has been taken into account during calculations of the metabolite concentrations. In order to limit evaporation during the batch cultures, the exhaust gases were passed into the exhaust condenser in which the moisture was removed and returned to the vessel. The cultures were performed in three replicates.

### Cloning and transformation protocols

All restriction enzymes were purchased from FastDigest Thermo Scientific (USA), and all of the digestions were performed according to standard protocols. The PCR reactions were set up using recommended conditions and Phusion high-fidelity DNA polymerase (Thermo Scientific). The ligation reactions were performed for 10 min at room temperature using T4 DNA Ligase (Thermo Scientific). The gel extractions were performed using the Gel Out gel extraction kit purchased from A&A Biotechnology (Poland). The *E. coli* minipreps were performed using the Plasmid Mini Kit (A&A Biotechnology). Transformation of *E. coli* strains was performed using standard chemical protocols [22]. Genomic DNA (gDNA) was extracted from *Y. lipolytica* using the Genomic Mini AX Yeast Spin kit (A&A Biotechnology, Poland). The obtained plasmids were digested with *Mss*I to create linear expression cassettes devoid of *E. coli* DNA and surrounded by *Y. lipolytica* rDNA for targeted integrations. The transformants were plated out on selective media [23] and were confirmed via gDNA extraction and three distinct PCR confirmations.

### Construction of overexpression plasmids

First, plasmid pAD carrying UAS1B_16_-TEF promoter was formed. Primers pAD-NotI-F and pAD-NotI-R amplified linear plasmid pAD-UTGut1 [23] but devoid of the *GUT1* gene. Next, the obtained PCR (7871 bp) product was digested with *Not*I and ligated, resulting in the pAD vector. The primers used in this study are listed in Additional file 1: Table S1.

After amplification of *Y. lipolytica* DNA with primers TKet-AscI-F and TKet-NheI-R, the 2458-bp PCR fragment was digested and cloned into the corresponding sites of pAD, resulting in pAD-TKL1. Gene *YALI0F15587*g, encoding transaldolase, was amplified, by primers Tald-AscI-F and Tald-NheI-R. The 1283-bp PCR product was digested and cloned into the corresponding sites of pAD, resulting in pAD-TAL1.

Gene *YALI0E22649*g was amplified by primers ZWF1-AscI-F and ZWF1-PmlI-R, the 1957-bp PCR product was digested with *Asc*I (*Sgs*I) and *Pml*I, and subsequently was cloned into the corresponding sites of pAD, yielding pAD-ZWF1.

Gene *YALI0B15598*g was amplified by primers GND1-AscI-F and GND1-NheI-R, the 2222-bp PCR product was digested with *Asc*I (*Sgs*I) and *Nhe*I, and subsequently was cloned into the corresponding sites of pAD, yielding pAD-GND1.

### Construction of hrGFP plasmids

First, the gene hr-GFP from plasmid phrGFP II-C (Agilent) using primers hrGFP-AscI-F/hr-NheI-R was amplified, digested with the enzymes, and cloned into previously digested pAD, yielding pAD-hrGFP. Construction of the promoter-gfp fusion plasmids P_TKL1_-gfp, P_TAL1_-gfp, P_ZWF1_-gfp, P_GND1_-gfp was carried out by amplifying the entire promoter region using primers Ptk-Bsp119I-F/Ptk-AscI-R, Ptal-Bsp119I-F/Ptal-AscI-R/, Pzwf-Bsp119I-F/Pzwf-AscI-R, Pgnd-Bsp119I-F/Pgnd-AscI-R. Subsequently, promoters were cloned into gel-extracted pAD (promotorless) digested with *Asc*I*/Bsp*119I, yielding P_TKL1_-gfp, P_TAL1_-gfp, P_ZWF1_-gfp, P_GND1_-gfp. The obtained plasmids were digested with *Mss*I and transformed into the strain *Y. lipolytica* AMM.

### RNA isolation and transcript quantification

The shake-flask cultures were grown for 24 h in Erythritol Synthesis Medium supplemented with glycerol (100 g/L) or in the Control Medium supplemented with glucose (100 g/L). Next, the cultures were collected and centrifuged for 5 min at 12,000*g*. The RNA was extracted using a Total RNA Mini Plus kit (A&A Biotechnology, Poland). Each sample was treated with DNAse I (Thermo Scientific) according to the manufacturer’s instructions. We measured RNA quantities using a Biochrom WPA Biowave II spectrophotometer (Biochrom Ltd., UK) equipped with a TrayCell (Hellma Analytics, Germany), and the samples were stored in a −80 °C freezer. We conducted cDNA synthesis using Maxima First Strand cDNA. Synthesis kits for RT-qPCR (Thermo Scientific) were used according to the manufacturer’s instructions. We carried out qRT-PCR analyses using a DyNAmo Flash SYBR Green qPCR Kit (Thermo Scientific) and the Eco Real-Time PCR System (Illumina, USA). Primers for RT-PCR were designed as follows: gene *TKL1* (*YALI0E06479g*) encoding the transketolase, gene *TAL1* (*YALI0F15987g*) encoding transaldolase, gene *GND1* (*YALI0B15598*g) encoding NADP^+^-dependent 6-phosphogluconate dehydrogenase, gene *ZWF1* (*YALI0E22649g*) encoding NADP^+^-dependent glucose-6-phosphate dehydrogenase were used as a template. Primers qTket-F and qTket-R bind to the first exon at 25 bp and to the second exon at 478 bp of the *TKL1* gene, respectively, resulting in a 111 bp qRT-PCR product. Next, primers qTald-F and qTald-R bind to the first exon at 42 bp and to the second exon at 388 bp of the *TAL1* gene, respectively, resulting in a 107 bp qRT-PCR product. Gene *GND1* possesses three introns; thus primer qGND1-F binds to the second exon at 257 bp, and primer qGND1-R binds to the second exon at 576 bp. The obtained PCR product in qRT-PCR is 105 bp. Primers qZWF1-F and qZWF1-R bind to the first exon at 5 bp and to the second exon at 595 bp of the *ZWF1* gene, respectively, resulting in a 151 bp qRT-PCR product. The results were normalized to the actin gene ACT-F/ACT-R and analyzed using the ddCT method [24]. Samples were analyzed in triplicate.

### Analytical methods

Samples (10 mL) from the cultures were centrifuged (10 min; 4 °C; 5500×*g*), harvested by filtration on 0.45-μm pore membranes and washed twice with distilled water. The biomass was determined gravimetrically after drying at 105 °C. The concentrations of the metabolites were determined using HPLC equipped with a HyperRez Carbohydrate H^+^ Column (Thermo Scientific, Waltham, MA) coupled to a UV (*λ* = 210 nm) (Dionex, Sunnyvale, USA) and a refractive index (RI) detector (Shodex, Ogimachi, Japan). The column was eluted with 25 mM of trifluoroacetic acid (TFA) at 65 °C and a flow rate of 0.6 mL/min.

### Fluorescence microscopy

Yeast cells were visualized using an Axio Scope A1 Zeiss microscope (Zeiss, Germany). A GFP filterset (Zeiss), operated at excitation of 470/40 nm, and emission of 525/50 nm, was used to detect green fluorescence. Microscopy data were stored using ZEN lite (Zeiss).

### Calculation of fermentation parameters

To consider medium dilution due to the addition of NaOH required for pH control, the amounts of erythritol and byproducts in the culture broth were used to calculate the mass yield of erythritol (*Y*_ERY_), and the volumetric erythritol productivity (*Q*_ERY_). The mass yield of erythritol (*Y*_ERY_) was expressed in g/g from glycerol and was calculated by the equation

$$Y_{\text{ERY}} = \, P/S.$$

The volumetric erythritol productivity (*Q*_ERY_) expressed in g/L/h was calculated from

$$Q_{\text{ERY}} = \, P/ \, V \cdot t,$$

where *P* is the amount of erythritol in the culture liquid at the end of cultivation (g); *S* is the total amount of glycerol consumed (g); *V* is the initial volume of culture liquid (l); and t is the fermentation time (h).

## Results and discussion

### Quantification of gene expression during erythritol synthesis

**Fig. 1:**
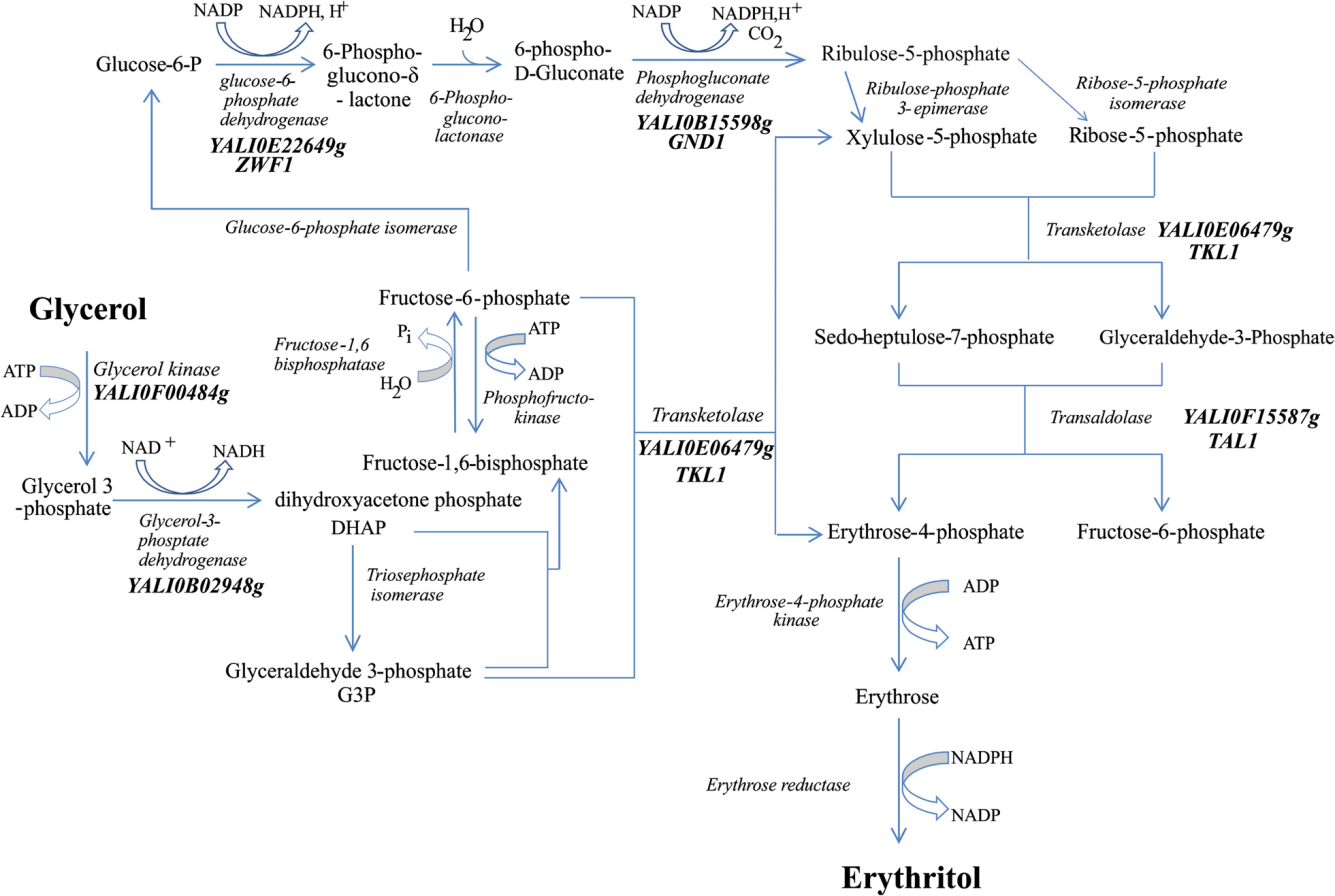
Overview of the principal metabolic pathway for erythritol synthesis in *Y. lipolytica*. Function of the genes names of which are given in *bold* characters was verified experimentally in this or in the previous study [23]

Production of erythritol by yeast is a well-known phenomenon; however, the metabolic pathway of this process for *Y. lipolytica* has never been characterized. Our previous study showed the hypothetical pathway of erythritol from glycerol in this oleaginous yeast [21]. In this study, we focused on four genes to investigate their role in erythritol biosynthesis. Here, we choose genes belonging to the pentose phosphate pathway (PPP), since it was shown that erythritol synthesis occurs via this pathway [25]. In this study, we selected genes encoding transketolase (*TKL1, YALI0E06479g*), transaldolase (*TAL1, YALI0F15587g*), NADP^+^-dependent 6-phosphogluconate dehydrogenase (*GND1, YALI0B15598g*), and NADP^+^-dependent glucose-6-phosphate dehydrogenase (*ZWF1, YALI0E22649g*). The first (oxidative) phase of PPP starts with dehydrogenation of glucose 6-phosphate, by 6-phosphogluconate dehydrogenase, resulting in NADPH and 6-phosphoglucono-δ-lactone synthesis (Fig. 1). Next, 6-phosphoglucono-δ-lactone is hydrolyzed by a specific lactonase to produce 6-phosphogluconate. Consequently, this sugar is oxidatively decarboxylated by 6-phosphogluconate dehydrogenase, yielding ribulose-5-phosphate along with cogeneration of NADPH. In the second, nonoxidative phase of PPP, transketolase converts ribose-phosphate and xylose-5-phosphate into glyceraldehyde 3-phosphate (GAP) and sedoheptulose 7-phosphate. Next, these compounds are catalyzed by transaldolase to form fructose 6-phosphate and erythrose 4-phosphate. Transketolase converts glyceraldehyde-3-phosphate with fructose-6-phosphate into xylulose-5-phosphate and erythrose-4-phosphate (E4P). The last step of erythritol synthesis is beyond PPP, and it is a reduction of erythrose to erythritol by erythrose reductase with codominant NADPH.

The standard carbon source for yeast *Y. lipolytica* is glucose, and it is commonly used in research and in industry. However, during our study, we observed that the level of erythritol synthesis by strain *Y. lipolytica* MK1 depends on the carbon source, and it is higher when glycerol is applied (Additional file 2). Probably it is due to higher osmotic stress caused by glycerol. Moreover, in agreement with another study, we observed that in the medium containing glucose as a carbon source, strain MK1 forms mycelium [26]. In medium with glycerol, only yeast cells were observed; this suggests that mycelia cannot synthesize erythritol efficiently. Therefore, to verify the activity of the PPP genes during erythritol synthesis, we compared the relative expressions of the mentioned genes under two different conditions. First, the strain MK1 was grown in the Erythritol Synthesis Medium (see “Methods”) in the baffled flasks; second, MK1 strain was grown in the Control Medium. Remarkably, immediately after 24 h, gene *TKL1* exhibited a sevenfold increase in expression over the control, and we also observed enhanced expressions for *ZWF1* and *TAL1*, by 3.0- and 1.8-fold respectively (Additional File 3: Figure S1).

**Fig. 2:**
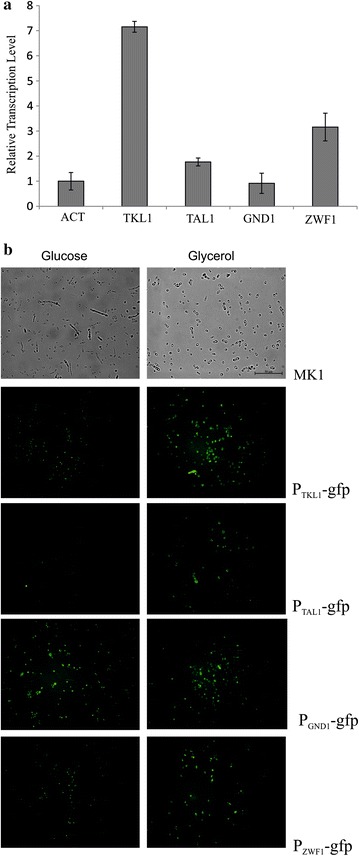
**a** Expression of genes in this study. Relative quantification of RNA transcript using RT-PCR; actin was used as a reference gene. Strain MK1 was grown in Erythritol Synthesis Medium or in the Control Medium. Samples were analyzed in triplicate, and the standard errors were estimated using Illumina Eco software. **b** The visualization of the P_TKL1_-gfp, P_TAL1_-gfp, P_GDN1_-gfp, and P_ZWF1_-gfp expressions in *Y. lipolytica* AMM; strain MK1 was used as a control. Strains were grown in the Control Medium (*left panels*) or in the Erythritol Synthesis Medium (*rights panels*). Pictures were taken at 72 h of the cultivation. DIC pictures are shown in Additional File 4: Figure S2

Interestingly, the expressions of the *GND1* gene remain at the same level under both conditions (Fig. 2a). To confirm the qRT-PCR results, we fused the promoters of the mentioned genes with hrGFP protein, to visualize their activities under both conditions. In agreement with previous experiment, we observed enhanced GFP expressions for *TLK1*, *ZWF1*, and *TAL1* in Erythritol Synthesis Medium (Fig. 2b). Strain AMM P_GND1_-*gfp* showed similar fluorescence under both conditions, suggesting that this dehydrogenase is active, independent of the applied carbon source. It is worth noting that we did not observe mycelium in the AMM-derived strains.

Previously, the activities of the enzymes encoded by these genes were studied for *Moniliella megachiliensis* (formerly *Trichosporonoides megachiliensis*) [20]. In that study, all enzymes belonging to PPP showed high activity during erythritol synthesis; however, transketolase was found as a crucial enzyme for the process. The results obtained in our study confirm that in *Y. lipolytica*, transketolase is expressed at an enhanced level in comparison with the other genes in the PPP.

### Overexpression of PPP genes in *Y. lipolytica*

First, we sought to verify the influence of the PPP genes’ overexpression on *Y. lipolytica* growth. Thus, we constructed overexpression cassettes containing the genes *TKL1*, *TAL1*, *ZWF1*, and *GND1* under a hybrid TEF promoter [27], and transformed the cassettes into the AMM strain, derived from MK1 [28], resulting in strains listed in Table 1.

**Fig. 3:**
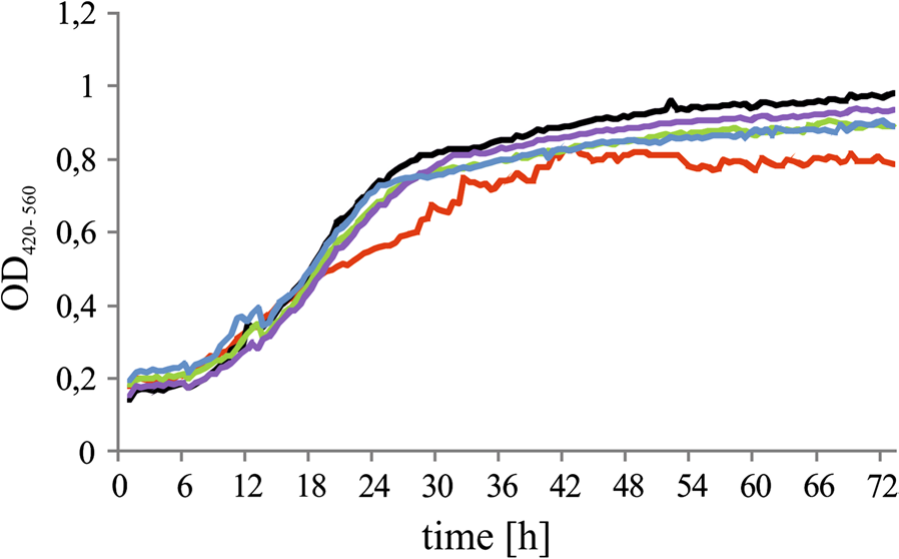
Growth curves of various *Y. lipolytica* strains: MK1 (*red*), AMM pAD-TKL1 (*black*), AMM pAD-TAL1 (*green*), AMM pAD-GND1 (*purple*), and AMM pAD-ZWF1 (*blue*). The strains were grown on a YNB/glycerol medium. Quintuple experiments were performed at 28 °C under constant agitation using Bioscreen C

Next, we monitored the growth of the engineered strains and the wild type in YNB media supplemented with glucose or glycerol. As seen in Fig. 3, all the engineered strains grew better on medium supplemented with glycerol than the control. In the first 24 h, the growth of the all strains was similar; however, after this period, when the maximum activity of the TEF promoter started, we noted significant enhancement in growth of the engineered strains. The control strain grew on YNB with glycerol in the similar way as in the previous study [11]. We did not observe any significant differences in growth between the engineered strains, either on YNB with glucose (data not shown) or on YNB with glycerol.

**Fig. 4:**
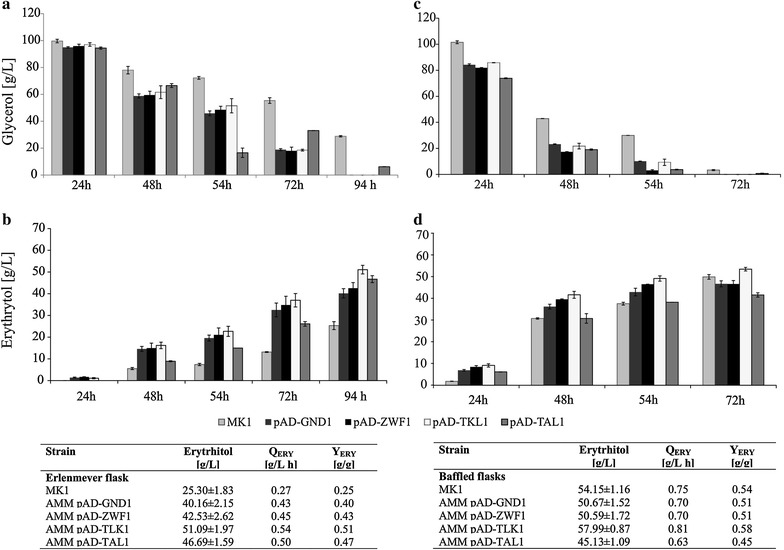
Results of the shake-flask experiment in the Erlenmeyer flasks (**a**, **b**) and in the baffled flasks (**c**, **d**). Parameters of erythritol synthesis by various *Y. lipolytica* strains in shake-flask experiments at the end of the process; *Q*_ERY_ erythritol productivity; *Y*_ERY_ erythritol yield. The cultures were performed in three biological replicates. The *error bars* represent the standard deviation. Tables summarize the parameters of erythritol synthesis at the end of the processes

Taking into account this result, the effects of *TKL1*, *TAL1*, *ZWF1,* and *GND1* overexpressions on erythritol synthesis were first assessed in the shake-flask experiment (Erlenmeyer flask). The conditions of the experiment were established previously [21]. In this study, we monitored two parameters: glycerol consumption and erythritol synthesis (Fig. 4a, b). After 24 h, we did not observe any significant differences in substrate assimilation or in erythritol biosynthesis. However, after 48 h, all the engineered strains showed elevated erythritol synthesis coupled with more rapid glycerol utilization. The most conspicuous effect was observed for the strain overexpressing the *TKL1* gene; this strain produced 51.09 ± 1.97 g/L of erythritol within 94 h. Erythritol titers for strains AMM pAD-TAL1, AMM pAD-ZWF1, and AMM pAD-GND1 were 46.69 ± 1.59, 42.53 ± 2.62, and 40.16 ± 2.15 g/L, respectively. The control strain produced only 25.30 ± 1.83 g/L. Although glycerol incorporation into the cell was not modified, we observed enhancement of glycerol utilization. In the cultures containing the overexpressing *TKL1*, *ZWF1*, and *GND1* strains, after 94 h, glycerol was totally depleted, whereas in the control strain, 28 g/L of glycerol was detected. This phenomenon might be a result of pulling the metabolism toward erythritol synthesis by overexpression of the genes involved in this pathway. This has an enormous impact on the erythritol productivity (*Q*_ERY_); the engineered strains achieved twofold higher productivity over the control strain. In addition, the yield of erythritol (*Y*_ERY_) was significantly improved (Fig. 4). The best impact on *Y*_ERY_ was noted for strain AMM pAD-TKL1, where the yield achieved was 0.51 g/g, whereas for the control strain, *Y*_ERY_ was only 0.35 g/g. It is worth noting that all the engineered strains showed enhanced capacity for erythritol biosynthesis from glycerol. Production of byproducts was also increased in the engineered strains; particularly, productions of mannitol and citric acid were significantly enhanced (Table 3). This is an effect of higher content of fructose 6-phosphate (in the PPP), which is a substrate for mannitol-1-phosphate and consequently for mannitol. The highest level of citric acid production was achieved by AMM pAD-GND1; this strain produced 18.54 ± 1.67 g/L of citric acid, which was threefold higher than that of the control strain (5.98 ± 0.10 g/L). Moreover, an enhanced level of citric acid is also caused by increased content of fructose 6-phosphate, which is one of the links of the chain in the Embden–Meyerhof–Parnas glycolytic pathway (EMP). The EMP is connected by pyruvate with the tricarboxylic acid (TCA) cycle. Therefore, overexpression of genes belonging to the PPP indirectly affect TCA.

**Table 3:** Side-metabolites of *Y. lipolytica* strains in shake-flask experiments in Erlenmeyer flasks

Time (h)	Strain	Arabitol (g/L)	Mannitol (g/L)	Citric acid (g/L)	α-Ketoglutaric acid (g/L)
24	MK1	0	0	0	0
	AMM pAD-GND1	0.20 ± 0.01	0.05 ± 0.01	1.13 ± 0.01	0
	AMM pAD-ZWF1	0.24 ± 0.03	0.01 ± 0.01	0.78 ± 0.01	0
	AMM pAD-TKL1	0	0	0.84 ± 0.01	0
	AMM pAD-TAL1	0	1.12 ± 0.03	0	0
48	MK1	0.40 ± 0.05	0.32 ± 0.06	2.60 ± 0.13	0.55 ± 0.02
	AMM pAD-GND1	0.93 ± 0.05	2.15 ± 0.02	9.94 ± 0.11	0.44 ± 0.05
	AMM pAD-ZWF1	1.33 ± 0.10	1.99 ± 0.23	8.58 ± 1.33	0.53 ± 0.08
	AMM pAD-TKL1	0.87 ± 0.10	1.52 ± 0.07	6.32 ± 2.18	0.77 ± 0.19
	AMM pAD-TAL1	1.04 ± 0.07	1.12 ± 0.03	5.05 ± 1.27	0.41 ± 0.04
54	MK1	0.43 ± 0.13	0.39 ± 0.09	3.05 ± 0.01	0.66 ± 0.06
	AMM pAD-GND1	1.12 ± 0.01	3.00 ± 0.09	11.50 ± 0.25	0.45 ± 0.20
	AMM pAD-ZWF1	1.58 ± 0.10	2.82 ± 0.15	10.33 ± 1.51	0.68 ± 0.15
	AMM pAD-TKL1	1.09 ± 0.01	2.48 ± 0.02	7.64 ± 2.51	1.11 ± 0.38
	AMM pAD-TAL1	1.52 ± 0.11	2.11 ± 0.45	5.85 ± 1.05	0.66 ± 0.13
72	MK1	0.64 ± 0.01	0.85 ± 0.04	4.54 ± 0.14	0.75 ± 0.05
	AMM pAD-GND1	1.32 ± 0.12	5.45 ± 0.22	16.24 ± 0.71	0.64 ± 0.30
	AMM pAD-ZWF1	2.01 ± 0.20	5.07 ± 0.01	13.62 ± 3.31	0.84 ± 0.32
	AMM pAD-TKL1	1.35 ± 0.05	4.41 ± 0.05	10.33 ± 2.82	1.60 ± 0.61
	AMM pAD-TAL1	1.70 ± 0.16	3.11 ± 0.65	7.92 ± 0.69	0.76 ± 0.1
94	MK1	0.89 ± 0.04	1.71 ± 0.16	5.98 ± 0.10	0.79 ± 0.08
	AMM pAD-GND1	1.79 ± 0.12	7.63 ± 0.48	18.54 ± 1.67	0
	AMM pAD-ZWF1	2.61 ± 0.16	7.02 ± 0.38	15.32 ± 4.28	0
	AMM pAD-TKL1	1.53 ± 0.39	6.66 ± 0.78	12.70 ± 2.18	0.31 ± 0.10
	AMM pAD-TAL1	2.30 ± 0.13	6.23 ± 0.36	11.16 ± 1.41	1.14 ± 0.52

It has been shown that oxygen concentration in the medium has a huge impact on erythritol synthesis by *M. megachiliensis* [20]. Therefore, to further characterize the engineered strains and explore their abilities of erythritol synthesis, the shake-baffled flask fermentation was conducted. Again, we monitored glycerol assimilation and erythritol synthesis (Fig. 4c, d). In agreement with our assumption, the process was much more efficient than when conducted in Erlenmeyer flasks. First, erythritol production started already after a few hours; therefore, at 24 h of growth, this polyol was detected for all tested strains, including the control. Total time of fermentation was only 72 h for all the engineered strains; for the control, residual glycerol was still detected (3.34 g/L). Increased aeration has the highest influence on the MK1 strain, because the titer of erythritol at the end of the process (51.15 ± 1.16 g/L) increased twofold in comparison with the previous experiment (25.3 ± 1.83 g/L). Nevertheless, changes of the condition also improved production of the *TKL1* overexpressing strain. Here, the erythritol titer was the highest among the tested strains, and it achieved 58.0 ± 0.87 g/L. This leads to enhancement of the process parameters; *Y*_ERY_ achieved 0.58 g/g and *Q*_ERY_ 0.81 g/L h. This is one of the highest amounts of erythritol titers obtained in the shake-flask experiments. A previously conducted experiment for *Y. lipolytica* in a shake-flask experiment showed a lower titer levels (20.03–35.53 g/L), *Y*_ERY_ (0.33–0.42 g/g), and *Q*_ERY_ (0.08–0.15 g/L h) [29]. Also in experiments using *Torula* sp. supplementation of culture media resulted in enhanced amount of erythritol titer, which was 56.6 g/L, whereas *Y*_ERY_ achieved was only 0.28 g/g [30].

Surprisingly, overexpressions of *ZWF1* and *GND1* at higher aeration resulted in higher production of erythritol than at low aeration (Fig. 4); however, after glycerol depletion, we noted rapid polyol assimilation. As a result, both stains showed a slightly lower erythritol titer than the control at the end of the process. These data suggest that both dehydrogenases might play a role in erythritol assimilation as a potential carbon source. Next, strain AMM pAD-TAL1 under high aeration conditions produced less erythritol than in low aeration condition. However, we noted that carbon flux was pushed toward citric acid synthesis. This strain produced 23.37 ± 0.92 g/L of citric acid in baffled flasks, which was a twofold improvement compared with Erlenmeyer flasks, where the citric acid level achieved was 11.16 ± 1.41 g/L. Moreover, it was the highest titer of citric acid among the tested strains (Table 4). This undesired effect for the downstream process can be eliminated by optimization of the fermentation conditions. It is worth noting that the optimization of fermentation conditions is important and has the same high impact on the process as metabolic engineering. Therefore, all genetic modifications should be coupled with the optimization of the fermentation conditions. In accordance with the aim of this study, and to further characterize the AMM pAD-TKL1 transformant and explore its erythritol production characteristics, a large-scale experiment was performed using a 5-L bioreactor.

**Table 4:** Side-metabolites of *Y. lipolytica* strains in shake-flask experiments in baffled flasks

Time (h)	Strain	Arabitol (g/L)	Mannitol (g/L)	Citric acid (g/L)	α-Ketoglutaric acid (g/L)
24	MK1	0.24 ± 0.03	0	2.38 ± 0.03	0.23 ± 0.01
	AMM pAD-GND1	0.15 ± 0.04	0.29 ± 0.01	8.05 ± 0.05	0.25 ± 0.00
	AMM pAD-ZWF1	0.35 ± 0.01	0.37 ± 0.05	9.00 ± 1.30	0.33 ± 0.04
	AMM pAD-TKL1	0.26 ± 0.01	0.32 ± 0.03	7.61 ± 0.21	0.47 ± 0.02
	AMM pAD-TAL1	0	3.20 ± 0.05	7.10 ± 0.50	0
48	MK1	0.77 ± 0.00	1.19 ± 0.01	9.53 ± 0.36	0.44 ± 0.01
	AMM pAD-GND1	0.70 ± 0.02	3.14 ± 0.02	14.26 ± 0.08	1.12 ± 0.08
	AMM pAD-ZWF1	1.05 ± 0.01	3.22 ± 0.13	15.37 ± 3.54	1.31 ± 0.24
	AMM pAD-TKL1	0.52 ± 0.00	2.43 ± 0.07	13.64 ± 0.54	2.10 ± 0.11
	AMM pAD-TAL1	0.93 ± 0.02	3.20 ± 0.05	17.03 ± 2.82	0.27 ± 0.19
54	MK1	0.76 ± 0.05	1.35 ± 0.27	9.99 ± 0.28	0.45 ± 0.01
	AMM pAD-GND1	0.74 ± 0.02	3.70 ± 0.05	15.60 ± 0.24	1.10 ± 0.08
	AMM pAD-ZWF1	1.08 ± 0.02	3.79 ± 0.13	16.64 ± 4.04	1.29 ± 0.24
	AMM pAD-TKL1	0.54 ± 0.00	2.87 ± 0.05	15.61 ± 0.54	2.04 ± 0.13
	AMM pAD-TAL1	1.16 ± 0.07	4.73 ± 0.15	13.67 ± 1.77	0.00 ± 0.00
72	MK1	0.71 ± 0.07	1.90 ± 0.08	10.69 ± 0.27	0.37 ± 0.02
	AMM pAD-GND1	0.75 ± 0.04	4.26 ± 0.10	15.95 ± 0.34	0
	AMM pAD-ZWF1	1.08 ± 0.10	4.27 ± 0.09	15.97 ± 3.83	0
	AMM pAD-TKL1	0.55 ± 0.01	3.40 ± 0.06	17.35 ± 1.60	0
	AMM pAD-TAL1	0.96 ± 0.25	4.72 ± 0.40	23.37 ± 0.92	0

### The effect of TKL1 overexpression in bioreactor fermentations

Given the results of the study, we sought to cultivate AMM pAD-TKL1 strain in a bioreactor fermentation, consistent with experiments previously conducted [23]. This strain was chosen since *TKL1* was found to be a crucial gene for erythritol production, and a transformant overexpressing *TKL1* showed the highest capacity for erythritol synthesis. During cultivation of *Y. lipolytica* on an industrial scale, one of the important issues is the high cost of energy consumption for continuous and intense agitation. This yeast involves high demand for oxygen; otherwise the fermentation parameters significantly decrease. In the shake-flask experiment, as we observed that low aeration did not have a negative influence on erythritol synthesis, we sought to conduct the fermentation process at lower agitation (500 rpm) than that applied in the standard procedures (800 rpm). The aeration was fixed at 0.8 L/min, and again as a control strain, MK1 was used.

**Fig. 5:**
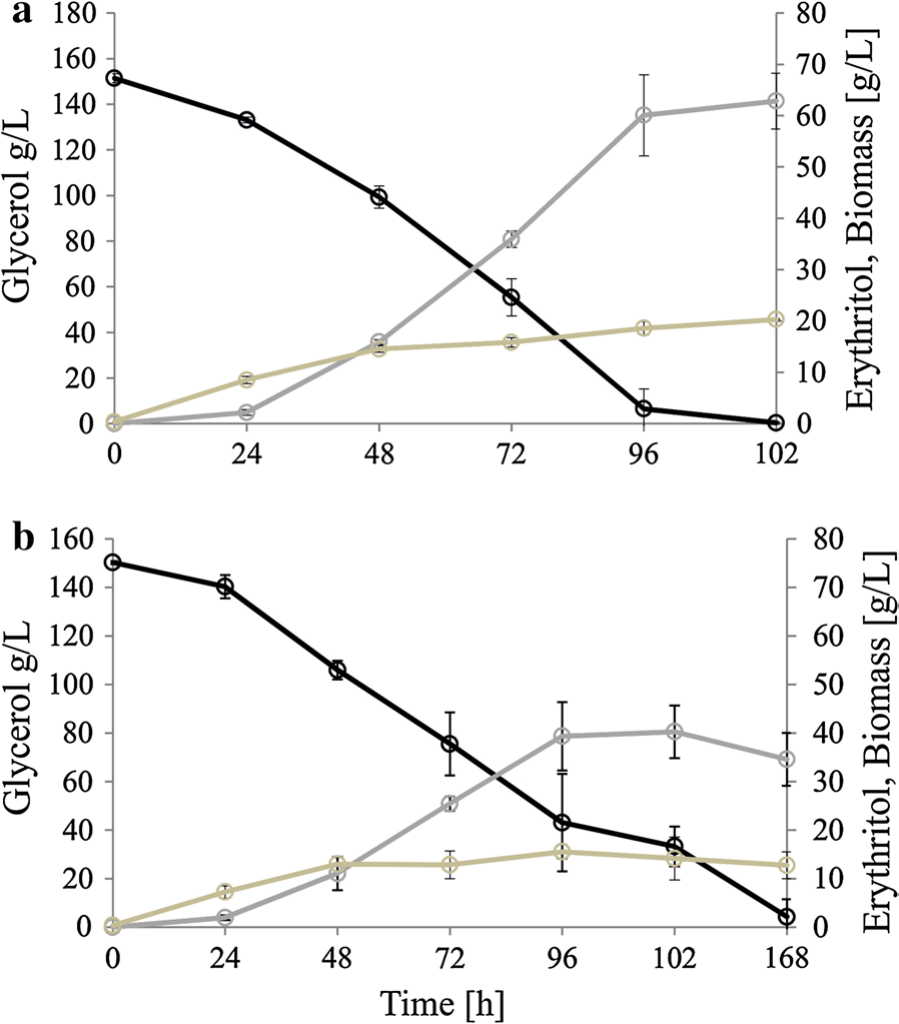
Batch bioreactor fermentations with strain overexpressing *TKL1* (**a**), and strain MK1 used as a control (**b**). Glycerol assimilation (*black line*), erythritol synthesis (*gray line*), and biomass production (*brown line*). The cultures were performed in three biological replicates. The *error bars* represent the standard deviation

In agreement with the other studies [31], the dissolved oxygen fell to below 1% after 24 h of growth. As seen in Fig. 5a for the strain overexpressing *TKL1,* low oxygen was not an obstacle to growth. At the end of the process, the biomass of AMM pAD-TKL1 strain reached 20 g/L, which is a standard biomass titer in the erythritol synthesis medium [23]. In contrast, the control strain produced a maximum 14.1 g/L biomass after 96 h of growth, and it decreased to 10 g/L at the end of the process. The first detection of erythritol was after 24 h for both stains, and we did not notice any difference between the two strains. However, after the next 24 h, when the highest activity of the *TEF* promoter started, we observed rapid production of erythritol for the engineered strain (Fig. 5a). The engineered strain produced 62.5 g/L of erythritol within 102 h, resulting in *Q*_ERY_ 0.62 ± 0.05 g/L h and *Y*_ERY_ 0.42 ± 0.05 g/g. Interestingly, production of the side-metabolites was significantly inhibited in comparison with the shake-flask experiment, and the maximum content of each of the undesired products did not exceed 5 g/L (Table 5). This effect was caused by the established and fully controlled fermentations process in the bioreactor; pH and the oxygen supply were maintained at constant levels. A similar effect during erythritol production was observed previously [21].

**Table 5:** Side-metabolites of *Y. lipolytica* strains in bioreactor study

Time (h)	Strain	Arabitol (g/L)	Mannitol (g/L)	Citric acid (g/L)	α-Ketoglutaric acid (g/L)
24	MK1	0	0	0	0
	AMM pAD-TKL1	0	0	0.02 ± 0.02	0.25 ± 0.07
48	MK1	0.35 ± 0.07	0	0.15 ± 0.2	0.70 ± 0.14
	AMM pAD-TKL1	0.76 ± 0.08	1.00 0.01	0.14 ± 0.10	1.21 ± 0.13
72	MK1	0.70 ± 0.01	0.35 ± 0.19	0.40 ± 0.17	0.90 ± 0.14
	AMM pAD-TKL1	1.21 ± 0.16	2.72 0.03	0.93 ± 0.52	2.93 ± 0.38
96	MK1	0.85 ± 0.07	1.20 ± 0.42	0.70 ± 0.57	0.90 ± 0.42
	AMM pAD-TKL1	1.33 ± 0.46	4.01 1.42	1.63 ± 0.67	4.07 ± 0.90
102	MK1	0.90 ± 0.14	1.15 ± 0.49	0.80 ± 0.51	0.95 ± 0.49
	AMM pAD-TKL1	1.40 ± 0.57	5.00 0.28	4.00 ± 1.97	3.95 ± 1.77
168	MK1	0.15 ± 0.21	0.25 ± 0.20	1.20 ± 0.28	0
	AMM pAD-TKL1	–	–	–	–

The low concentration of side-metabolites is important for industrial applications, since it simplifies the purification process and consequently reduces production costs. The engineered strain assimilated glycerol within 102 h of fermentation, whereas the control strain was not able to deplete glycerol during 168 h of cultivation. Moreover, biosynthesis of erythritol was limited compare to the engineered strain, and the titer was 37.3 g/L at the end of the process, resulting in *Q*_ERY_ 0.27 g/L h and *Y*_ERY_ 0.25 g/g. Due to weak growth of the control strain, production of byproducts was highly limited (Table 5).

The process conducted for the control strain at low agitation was inefficient and time consuming compares with the previous studies [5, 21, 32]. These data confirm that oxygen limitation has a huge impact on erythritol synthesis by *Y. lipolytica*. These results showed that transketolase plays a crucial role in erythritol synthesis and its overexpression allows for robust growth independent of oxygen supply. The engineered strain AMM pAD-TKL1 synthesized erythritol efficiently in a short period of time at a low oxygen concentration. It has a huge potential for industrial application.

## Conclusions

Until now, the metabolic pathway of erythritol synthesis in *Y. lipolytica* has been never described. In this study, we showed that erythritol production in *Y. lipolytica* occurs via the pentose phosphate pathway (PPP) and the gene *YALI0E06479*g encoding transketolase plays a crucial role in this process. Moreover, gene *YALI0E22649g* encoding glucose-6-phosphate dehydrogenase (*ZWF1*) provides the reducing agent which is required in the last step of the process. Overexpression of the genes belonging to the PPP enhances the level of erythritol titer. An increased level of *TKL1* transcription results in significantly higher erythritol synthesis under conditions of oxygen limitation. During the shake-flask experiment, the engineered strain increased erythritol production twofold over the control. Over the course of the bioreactor study, strain AMM pAD-TKL1 produced 67% more erythritol than the control, and production of the by-product was significantly inhibited. Moreover, these results were obtained using a low-cost medium in which the single carbon source was glycerol and at low agitation; this situation is beneficial for downstream processing.

## Additional files

**Additional file 1: Table S1.** The primer list used in this study.

**Additional file 2.** Production of erythritol and carbon source utilization in the baffled flasks conducted in the Erythritol Synthesis medium and in the Control Medium.

**Additional file 3: Figure S1.** Quantification of gene expression during erythritol synthesis by the strain MK1 strain at 24 h of growth in Erlenmeyer flasks. Samples were analyzed in triplicate and the standard errors were estimated using Illumina Eco software.

**Additional file 4: Figure S2.** The visualization of the PTKL1-gfp, PTAL1-gfp, PGDN1-gfp and PZWF1-gfp expression in *Y. lipolytica* AMM. Strains were grown in the Control Medium (left panels) or in the Erythritol Synthesis Medium (rights panels). Pictures were taken at 72 h of the cultivation.

## Abbreviations

*TKL1*: gene *YALI0E06479g* encoding transketolase
*TAL1*: gene *YALI0F15587g* encoding transaldolase
*ZWF1*: gene *YALI0E22649g* encoding glucose-6-phosphate dehydrogenase
*GND1*: gene *YALI0B15598g* encoding 6-phosphogluconate dehydrogenase
