# Supplementary material for: Functional overexpression of genes involved in erythritol synthesis in the yeast Yarrowia lipolytica
Source: Biotechnol Biofuels. 2017 Mar 24;10:77. doi: 10.1186/s13068-017-0772-6 (PMC5366165; doi:10.1186/s13068-017-0772-6)
Supplement: Supplementary file 1 — Additional file 1: Table S1. The primer list used in this study. [file 13068_2017_772_MOESM1_ESM.pdf]

Additional File 2 Table S1. The primer list used in this study

Name	Sequence (5'-3')
pAD-NotI-F	ATAGCGGCCGCGCGCGGCTAGCATTTAAATCAC
pAD-NotI-R	ATAGCGGCCGCGGCGCGCCTTTGAATGATTC
TKet-AscI-F	GATGGCGCGCCATGGCTCCCCAATTTTC
TKet-NheI-R	GCGGCTAGCGGCAACCCACTTAGAC
Tald-AscI-F	CATGGCGCGCCATGTCTTCCAACTCTCTTGAACAGCTTAAG
Tald-NheI-R	GCAGCTAGCCAAAGAGACGCCTAAG
ZWF1-AscI-F	CTAGGCGCGCCATGACTGGCACCTTAC
ZWF1-PmlI-R	GCACACGTGTCACGAGGAGCCCTTGGTGAC
GDN1-AscI-F	CCGGGCGCGCCATGACTGACACTTCAAACAT
GDN1-NheI-R	CGCGCTAGCTTAAGCATCGTAAGTGGAAGAAG
ACT-F	GAGTCACCGGTATCGTTC
ACT-R	GCGGAGTTGGTGAAAGAG
qGDN1-F	TCGCTGACATTGCCCTCATC
qGDN1-R	TGGAGGTGGTTCGGTTGTAG
qZWF1-F	CTGGCACCTTACCCAAGTTC
qZWF1-R	CGTGCGTAGCCGATGATTTC
qTket-F	GACGAGACTGCCATCAAC
qTket-R	CAGAACGTGGGCAACAGGAG
qTald-F	CACCGTTGTCACCGACACTG
qTald-R	ACTCGGGCTTCTTGGAAGCG
hrGFP-AscI-F	TATGGCGCGCCATGGTGAGCAAGCAGATC
hr-NheI-R	GCAGCTAGCCTGCAGAATTCCTATTAC
Ptk- Bsp119I-F	CGTTTCGAACGGTGGGTGATGAGAC
Ptk-AscI-R	TAAGGCGCGCCAGCCATTTTGAAAGTTGTC
Ptal-Bsp119I-F	GCGTTCGAAATCTGACGGCAGAGTG
Ptal-AscI-R	GCAGGCGCGCCCATTGTGTGTTTGTGTGTAA
Pzwf-Bsp119I-F	GCATTCGAAGCGCCAACTTTCATGC
Pzwf-AscI-F	TATGGCGCGCCAGTCATGGTGATGTATG
Pgdn-Bsp119I-F	GCCTTCGAACCGAGATTTGTGACGTTTG
Pgdn-AscI-R	TACGGCGCGCCCATTGTGTGATGGTGTCGTTA
