# Supplementary material for: Functional overexpression of genes involved in erythritol synthesis in the yeast Yarrowia lipolytica
Source: Biotechnol Biofuels. 2017 Mar 24;10:77. doi: 10.1186/s13068-017-0772-6 (PMC5366165; doi:10.1186/s13068-017-0772-6)
Supplement: Supplementary file 2 — Additional file 2. Production of erythritol and carbon source utilization in the baffled flasks conducted in the Erythritol Synthesis medium and in the Control Medium. [file 13068_2017_772_MOESM2_ESM.pdf]

Additional File 1. Production of erythritol and carbon source utilization in the baffled flasks conducted in the Erythritol Synthesis medium and in the Control Medium.

**Medium**	**Erythritol [g/L]**		
	24h	48h	72h
Erythritol synthesis Medium	2.51±0.84	38.1±2.32	41.14±3.68
Control Medium	0	15.24±2.06	20.12±0.19
	**Glycerol/Glucose [g/L]**		
	24h	48h	72h
Erythritol synthesis Medium	96.15±1.68	29.73±3.00	5.00±0.7
Control Medium	89.74±2,94	43.64±1,27	7.99±1,06
