# Supplementary material for: Functional overexpression of genes involved in erythritol synthesis in the yeast Yarrowia lipolytica
Source: Biotechnol Biofuels. 2017 Mar 24;10:77. doi: 10.1186/s13068-017-0772-6 (PMC5366165; doi:10.1186/s13068-017-0772-6)

Additional File 3. Figure S1. Quantification of gene expression during erythritol synthesis by the strain MK1 strain at 24 hours of growth in Erlenmeyer flasks. Samples were analyzed in triplicate and the standard errors were estimated using Illumina Eco software .

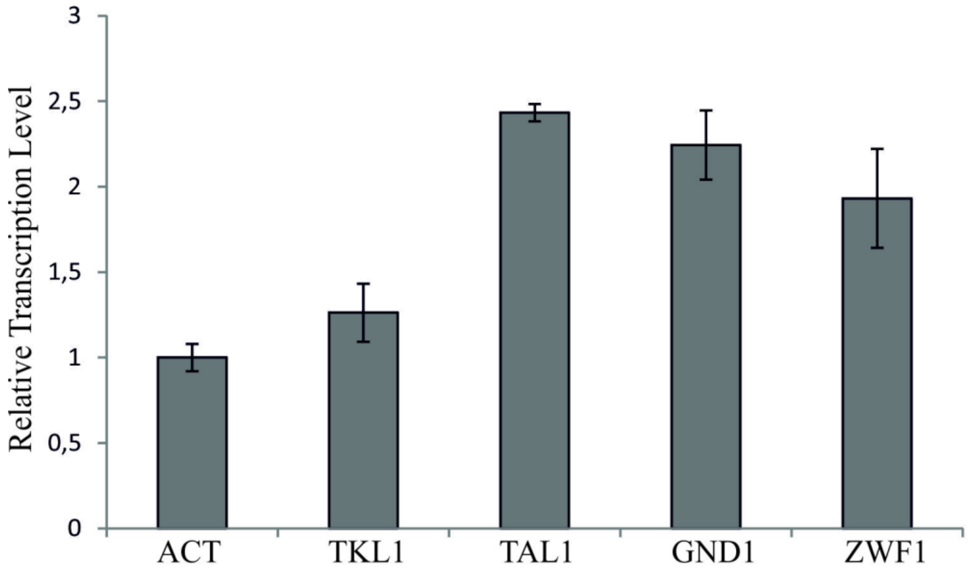

Supplement: Supplementary file 3 — Additional file 3: Figure S1. Quantification of gene expression during erythritol synthesis by the strain MK1 strain at 24 h of growth in Erlenmeyer flasks. Samples were analyzed in triplicate and the standard errors were estimated using Illumina Eco software. [file 13068_2017_772_MOESM3_ESM.pdf]
