# Supplementary material for: Functional overexpression of genes involved in erythritol synthesis in the yeast Yarrowia lipolytica
Source: Biotechnol Biofuels. 2017 Mar 24;10:77. doi: 10.1186/s13068-017-0772-6 (PMC5366165; doi:10.1186/s13068-017-0772-6)

Additional File 4 Figure S2. The visualization of the PTKL1-gfp, PTAL1-gfp, PGDN1-gfp and PZWF1-gfp expression in *Y. lipolytica* AMM. Strains were grown in the Control Medium (left panels) or in the Erythritol Synthesis Medium (rights panels). Pictures were taken at 72 hours of the cultivation.

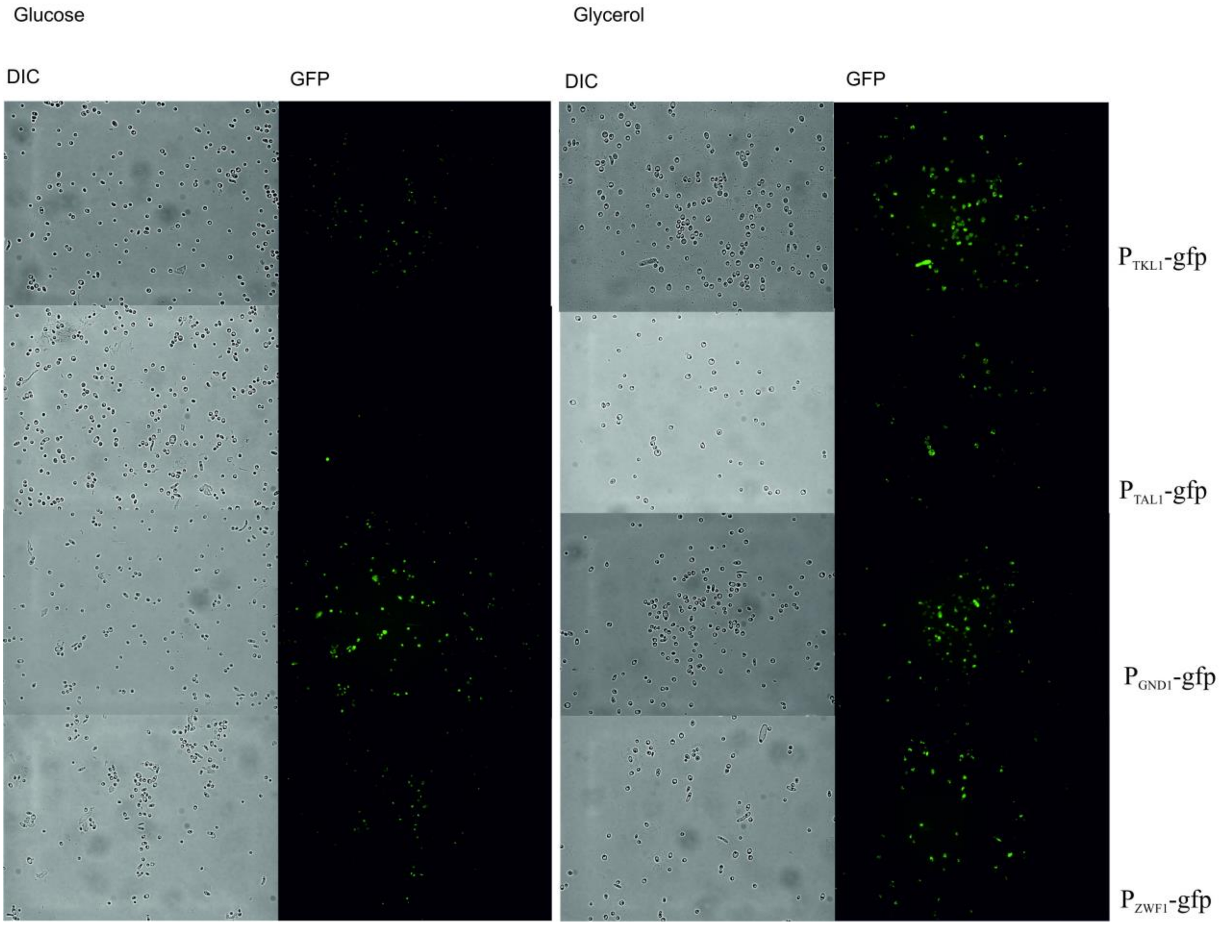

Supplement: Supplementary file 4 — Additional file 4: Figure S2. The visualization of the PTKL1-gfp, PTAL1-gfp, PGDN1-gfp and PZWF1-gfp expression in Y. lipolytica AMM. Strains were grown in the Control Medium (left panels) or in the Erythritol Synthesis Medium (rights panels). Pictures were taken at 72 h of the cultivation. [file 13068_2017_772_MOESM4_ESM.pdf]
